# Influence of Local Muscle Cooling on Mitochondrial-Related Gene Expression at Rest

**DOI:** 10.3390/ijerph191912028

**Published:** 2022-09-23

**Authors:** Larry Robins, Monica Kwon, Mark L. McGlynn, Alejandro M. Rosales, Elizabeth J. Pekas, Christopher Collins, Song-Young Park, Dustin R. Slivka

**Affiliations:** 1 School of Health and Kinesiology, University of Nebraska at Omaha, Omaha, NE 68182, USA; 2 School of Integrated Physiology and Athletic Training, University of Montana, Missoula, MT 59812, USA

**Keywords:** temperature, PGC1-*α*, GABPA, mRNA, cold

## Abstract

The purpose of this study was to determine the impact of localized cooling of the skeletal muscle during rest on mitochondrial related gene expression. Thermal wraps were applied to the *vastus lateralis* of each limb of 12 participants. One limb received a cold application (randomized) (COLD), while the other did not (RT). Wraps were removed at the 4 h time point and measurements of skin temperature, blood flow, and intramuscular temperature were taken prior to a muscle biopsy. RT-qPCR was used to measure expression of genes associated with mitochondrial development. Skin and muscle temperatures were lower in COLD than RT (*p* < 0.05). Femoral artery diameter was lower in COLD after 4 h (0.62 ± 0.05 cm, to 0.60 ± 0.05 cm, *p* = 0.018). Blood flow was not different in COLD compared to RT (259 ± 69 mL·min^–1^ vs. 275 ± 54 mL·min^–1^, *p* = 0.20). *PGC-1α B* and *GABPA* expression was higher in COLD relative to RT (1.57-fold, *p* = 0.037 and 1.34-fold, *p* = 0.006, respectively). There was no difference (*p* > 0.05) in the expression of *PGC-1α*, *NT-PGC-1α*, *PGC-1α A*, *TFAM*, *ESRRα*, *NRF1*, *GABPA*, *VEGF*, *PINK1*, *PARK 2*, or *BNIP3-L*. The impact of this small magnitude of difference in gene expression of *PGC-1α B* and *GABPA* without alterations in other genes are unknown. There appears to be only limited impact of local muscle cooling on the transcriptional response related to mitochondrial development.

## 1. Introduction

Endurance exercise plays a key role in mitochondrial homeostasis stimulating the continuous development (biogenesis) and breakdown (mitophagy) of the mitochondrial complex. Mitochondrial dysfunction is characteristic of diseases such as aging [1], obesity [2], and Parkinson’s disease [3]. Gene expression related to mitochondrial development is altered as part of the exercise response [4]. In addition to exercise, environmental and local temperature interventions may have the potential to alter gene expression related to mitochondrial development and aid in the treatment/prevention of mitochondrial dysfunction.

There are several nuclear encoded genes that play a role in the development of mitochondria. *Peroxisome proliferator-activated receptor gamma coactivator 1 alpha* (*PGC-1α*) and its isoforms (*PGC-1α A*, *PGC-1α B*, *NT-PGC-1α*) regulate mitochondrial biogenesis by binding to and co-activating *nuclear respiratory factor 1* (*NRF1*) and *nuclear factor*, *erythroid 2 like 2* (*NFE2L2*). *NRF1* and *NFE2L2* are responsible for the transcriptional activation of the mitochondrial DNA, leading to increased expression of *transcription factor A mitochondrial* (*TFAM*). *TFAM* regulates the transcription of mitochondrial DNA and translocates to the mitochondria where it stimulates gene expression [5,6]. *PGC-1α* also regulates *estrogen-related receptor α* (*ESRRα*) and *vascular endothelial growth factor* (*VEGF*) [7]. *ESRRα* regulates the genes associated with cellular energy production and mitochondrial biogenesis [6]. *VEGF* primarily induces angiogenesis but may also play a role in regulating a variety of mitochondrial biogenesis genes [6]. Mitochondrial biogenesis occurs along with mitophagy for net mitochondrial development. Mitophagy aids in promoting the breakdown of faulty and damaged mitochondria. *BNIP3-like* (*BNIP3-L*) recruits *GABARAP-L1* to damaged mitochondria for mitochondrial clearance [8]. *Bcl-2/adenovirus E1B interacting protein* (*BNIP3*) plays a role in the formation of autophagosomes [9]. *PTEN-induced putative protein kinase 1* (*PINK 1*) and *Parkin RBR E3 Ubiquitin Protein Ligase* (*PARK 2*) selectively concentrate on depolarized or damaged mitochondria [10].

Combinations of environmental temperature, local temperature, rest, and/or exercise have shown differential effects on mitochondrial related gene expression. Exercise [11,12], and even more so exercise in the ambient cold [13,14,15,16], are commonly found to stimulate *PGC-1α* gene expression. Ambient cold exposure at rest has shown no alterations in mitochondrial-related gene expression [17]. However, exercise combined with local cold application displays an inhibitory/blunting [18] effect when applied during exercise, but no effect on mitochondrial related gene expression when cooling occurs after exercise [19]. The interpretation of these findings could be enhanced by the isolation of local cooling under resting conditions alone. The application of cold directly to the skeletal muscle at rest may provide a different response than during exercise, after exercise, or with ambient cold exposure. The lower muscle temperatures that may be achieved without exercise and with direct cold application may provide a potent stimuli. Indeed, muscle glycogen resynthesis can be diminished when direct cooling is applied to the skeletal after exercise [20]. The effects of local cold application to the skeletal muscle independent of exercise may help contextualize the impact of muscle cooling on mitochondrial related gene expression.

The effects of local cold application independent of exercise on gene expression has received relatively little attention. Therefore, the purpose of this study is to determine the impact of localized cooling of the skeletal muscle during rest on gene expression related to mitochondrial homeostasis. By adding localized cold to the muscle, we can observe how decreasing muscle temperature impacts mitochondrial related gene expression without an exercise or other heat generating stimuli.

## 2. Materials and Methods

Twelve, apparently healthy subjects (10 males, 2 females) participated in this study. To be considered “apparently healthy”, subjects needed to be free from any signs or symptoms related to COVID and health conditions that would impact their ability to complete the study. Participants were informed of all the procedures and risks before signing an Institutional Review Board approved consent document (University of Nebraska Medical Center, protocol #388-20-FB) that conformed to ethical principles set forth by the declaration of Helsinki.

### 2.1. Study Design

Subjects were instructed to arrive at the laboratory following an overnight fast. Upon arrival, height (Seca Stadiometer; Hamburg, Germany) and weight (Befour Digital Scale; Saukville, WI, USA) were measured in exercise attire. The subjects rested in a supine position for 10 min before body composition was assessed via bioelectrical impedance analysis (Body Composition Analyzer S10, InBody; Seoul, Korea). Thermal wraps (1ACS266, Zamar Medical; Poreč, Croatia) were then applied to each thigh for 4 h. Legs were randomized to account for leg-to-leg differences. Circulating fluid in the thermal wrap of the experimental leg was cooled to 10 °C (COLD) while the other remained at room temperature with no fluid circulation (RT) as a control. Therefore, the experimental and control trials were accommodated in one visit. A standardized meal of commercially available pre-packaged foods (624 ± 56 Kcal; 88 ± 6 g of Carbohydrate, 27 ± 1 g of Protein, 19 ± 3 g of Fat) was provided after 30 min to ensure subjects were in a relatively similar nutritional state while also providing essential nutrients for optimal gene expression. Subjects were instructed to consume the meal within 30 min. Wraps were removed at the 4 h time point and measurements for skin temperature, arterial hemodynamics, and intramuscular temperature were taken prior to muscle biopsies on each leg.

### 2.2. Skin Temperature

Skin temperature was measured immediately after the wraps were removed, using a laser thermometer accurate to ±1.5 °C (FLUKE 62 Max+, Fortive Corp., Everett, WA, USA) on the surface of the skin over each *vastus lateralis* (VL). An image of both legs was also taken with a thermal camera accurate to ±2 °C (FLIR C3, FLIR Systems Inc., Wilsonville, OR, USA). Later analysis of the image utilized FLIR Tools (FLIR C-7200, FLIR Systems Inc., Wilsonville, OR, USA) to measure average skin temperatures of each VL. An ellipse, approximately 329.7 cm^2^ (7 cm × 15 cm), was calculated for best coverage of the VL surface and the average skin temperature of this area was recorded.

### 2.3. Arterial Hemodynamics

Average blood velocity and vessel diameter of the femoral artery (FA) of each leg were measured using ultrasound (Terason uSmart 3300, Terason Division Teratech Corporation, Burlington, MA, USA) by the same researcher for measurement consistency. Second-by-second velocity was collected using a frequency of 5 MHz with an isonation angle of 60° which was used to calculate average velocity (Vmean). Blood flow in mL·min^−1^ was calculated using Vmean ∗ π (FA diameter/2)^2^ ∗ 60 [21,22], where V is blood velocity and FA is Femoral Artery diameter. Arterial diameter was quantified at a perpendicular angle along the central axis of the FA using the ultrasound system [22]. Doppler ultrasound data (FA diameter and blood flow) were analyzed to determine mean antegrade shear rate. Mean antegrade shear rate is the process of blood flowing forward along the arterial wall and was calculated as (4 ∗ Vmean/FA diameter).

### 2.4. Intramuscular Temperature and Biopsies

The incision site at the VL (~10 cm proximal to the patella and ~5 cm lateral from the center of the thigh) was anesthetized with ~3 mL of 1% lidocaine injected under the surface of the skin and surrounding muscle fascia. The anesthetized site was sterilized with betadine before a small incision was made at the skin and into the fascia. A hypodermic thermocouple accurate to ±0.1 °C (MT-26/4HT, Physitemp Instruments LLC., Clifton, NJ, USA) was inserted up to ~4 cm into the incision. The thermocouple orientation and depth was similar to the muscle biopsy. That is, perpendicular to the incision and into the VL muscle belly. Temperature was recorded when the readings stabilized. After thermocouple removal, a 5 mm Bergström percutaneous muscle biopsy needle was inserted through the incision and into the VL muscle belly. A muscle sample was obtained with the aid of suction [23]. Muscle from the COLD limb was collected before the RT limb to minimize leg warming from the ambient environment. The experimental muscle sample was collected 17 ± 8 min after the wraps were removed and the control muscle was collected 27 ± 8 min after the wraps were removed. Following removal of excess blood, connective tissue, and fat, muscle samples (~60–80 mg) were quickly immersed in All-Protect preservation solution (All-Protect Tissue Reagent, Qiagen, Hilden, Germany). Samples were placed at 3 °C overnight before storage at −30 °C until analysis. After sample collection, incision sites were treated with antibiotic ointment and closed with steri-strips and bandages.

### 2.5. mRNA Analyses

Muscle mRNA of specific genes was determined using quantitative real-time reverse transcriptase polymerase chain reaction (qRT-PCR). Skeletal muscle samples were homogenized in 800 μL of Trizol (Invitrogen, Carlsbad, CA, USA) using an electric homogenizer (Fisher Scientific Homogenizer 150, Thermo Fisher Scientific, Waltham, MA, USA). Samples were incubated at room temperature for 5 min, 160 μL of chloroform was added, and the tubes were shaken by hand for 15 s. After another short incubation at room temperature (2–3 min) samples were centrifuged at 12,000× *g* for 15 min. The aqueous phase was transferred to a fresh tube and 400 μL of isopropyl alcohol was added prior to overnight incubation at −20 °C to precipitate mRNA. The following day, samples were centrifuged at 12,000× *g* for 10 min at 4 °C and the mRNA was washed in 75% ethanol. Samples were then dryed and the suspended in 30 μL of RNA storage solution. RNA concentration (325.52 ± 23.18 ng/μL) was measured with a nano-spectrophotometer (nano-drop ND-1000, Wilmington, DE, USA). RNA integrity (RIN: 8.47) was also quantified (Agilent 2100 Bio-Analyzer, Santa Clara, CA, USA) to ensure high quality intact RNA.

Superscript-first-strand synthesis system for qRT-PCR (Superscript IV, Invitrogen) was used to convert RNA to complementary DNA (cDNA). Each sample within a subject was analyzed in triplicate and adjusted to contain a standard cDNA concentration (5.62 ng/μL) by dilution using RNase free water. Each qRT-PCR 20-μL reaction volume contained 1 μL probe and primer mix (PrimeTime qRT-PCR assay, Integrated DNA technologies), 10 μL PrimeTime Gene Expression Master Mix (Integrated DNA technologies), 5 μL deionized water, and 4 μL of sample cDNA. Samples were analyzed using an Agilent Technologies Aria Mx real time PCR detection system (Agilent Technologies Inc.) running 1 cycle at 95 °C for 3 min, then 40 cycles of 95 °C for 5 s, and 60 °C for 10 s. Quantification of mRNA for genes of interest was calculated using the 2^−∆∆ct^ method [24] relative to stable reference genes (1.0-fold change). The geometric mean of the following 4 reference genes was used as the stable reference point: *beta-actin* (*ACTB*), *β2-microglobulin* (*B2M*), *ribosomal protein S18* (*RPS18*), and *glyceraldehyde-3 phosphate dehydrogenase* (*GAPDH*) for each participant. Stability was analyzed on an individual subject (both control and experimental samples) as opposed to the whole group to ensure each subject was represented as an independent observation and thus meeting assumptions associated with statistical analysis. NormFinder [25] was used to examine reference gene stability. If a reference gene, for a given individual, had a stability value of >0.15 it was removed from analysis and the geometric mean of the remaining reference genes were used. The muscle samples were analyzed for genes associated with biogenesis: *PGC-1α*, *NT-PGC-1α*, *PGC-1α A*, *PGC-1α B*, *transcription factor A mitochondrial* (*TFAM*), *estrogen-related receptor α* (*ESRRα*), *nuclear respirator factor 1* (*NRF1*), *GA binding protein transcription factor alpha* (*GABPA*), and *vascular endothelial growth factor* (*VEGF*). The muscle samples were also analyzed for genes associated with mitophagy: (*PTEN-induced putative protein kinase 1* (*PINK1*), *Parkin RBR E3 Ubiquitin Protein Ligase* (*PARK 2*), *Bcl-2/adenovirus E1B interacting protein* (*BNIP3*), and *BNIP3-like* (*BNIP3-L*)). Genetic sequences are referenced in Table 1.

### 2.6. Statistical Analysis

Two-tailed paired samples t-tests were used to determine differences between COLD and RT for vessel diameter, blood flow measurements, and gene expression data. Gene expression data was log transformed to ensure a normal distribution. Statistical tests were run in Excel. Significance was set at *p* < 0.05. Data are expressed as mean ± SD, unless reported as otherwise. Post hoc power analysis (G*Power, v3.1, Heinrich Heine Universität Düsseldorf, Düsseldorf, Germany) using mean intramuscular temperature differences revealed an achieved power of 1.0.

## 3. Results

The twelve subjects (10 males, 2 females) were 27 ± 6 years of age, 179 ± 9 cm tall, 82.8 ± 13.0 kg, and had a body fat percentage of 18.4 ± 7.1.

### 3.1. Temperature

Single point skin temperature measured via laser thermometer was lower after COLD than RT (13.2 ± 1.0 °C vs. 34.8 ± 0.9 °C; *p* < 0.001). Similarly, mean thermal camera skin temperature measurements were lower after COLD than RT (13.8 ± 0.9 °C vs. 34.9 ± 1.1 °C, *p* < 0.001). No differences were noted between laser thermometer and thermal camera measurements (*p* = 0.12 for COLD, *p* = 0.93 for RT). Intramuscular temperature measured via hypodermic thermocouple was lower in COLD than RT (20.5 ± 1.3 °C vs. 35.6 ± 0.8 °C, *p* < 0.001).

### 3.2. Arterial Hemodynamics

FA blood velocity was not different between limbs (*p* = 0.45). Arterial diameter was smaller in COLD than RT (*p* = 0.02). The resulting blood flow was not statistically different in COLD compared to RT (*p* = 0.20). Additionally, antegrade shear rate was not different between conditions (*p* = 0.98). See Table 2.

### 3.3. Gene Expression

**Figure 1:**
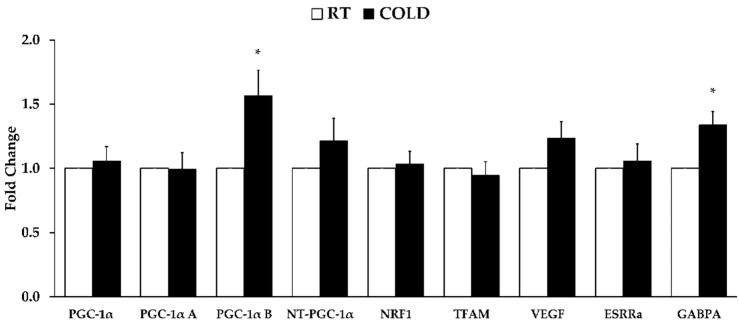
Skeletal muscle mitochondrial biogenesis-related messenger RNA expression 4 h post cold application. Shown genes include: *Peroxisome proliferator-activated receptor gamma coactivator 1 alpha* (*PGC-1α*), *PGC-1α A*, *PGC-1α B*, *Truncated PGC-1α* (*NT-PGC-1α*), *nuclear respiratory factor 1* (*NRF1*), *transcription factor A mitochondrial* (*TFAM*), *vascular endothelial growth factor* (*VEGF*), *estrogen-related receptor α* (*ERRα*), and *GA-binding protein transcription factor Alpha Subunit* (*GABPA*). Data are mean ± SE. * *p* < 0.05 from RT.

**Figure 2:**
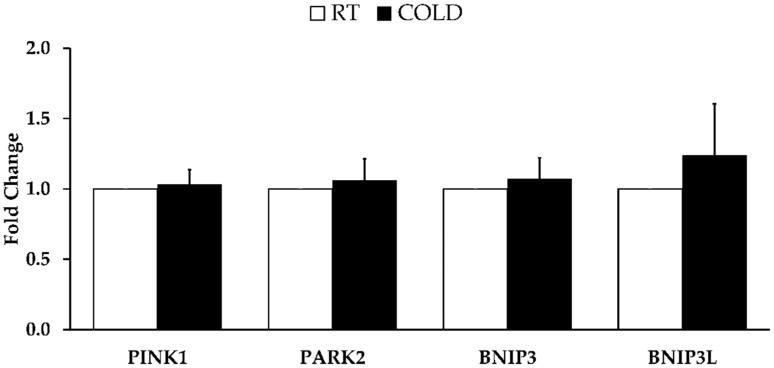
Skeletal muscle mitophagy-related mRNA expression 4 h post cold application. Shown genes include: *PTEN-induced putative protein kinase 1* (*PINK1*), *Parkin RBR E3 Ubiquitin Protein Ligase* (*PARK 2*), *Bcl-2/adenovirus E1B interacting protein* (*BNIP3*), and *BNIP3-like* (*BNIP3-L*). Data are mean ± SE.

*PGC-1α B* and *GABPA* were higher after COLD than RT (*p* = 0.037 and *p* = 0.006, respectively). The other genes related to mitochondrial biogenesis were not different between limbs (*PGC-1α*, *p* = 0.928; *PGC-1α A*, *p* = 0.463; *NT-PGC-1α*, *p* = 0.507; *NRF1*, *p* = 0.396; *TFAM*, *p* = 0.354; *VEGF*, *p* = 0.200; *ESSRα*, *p* = 0.894). See Figure 1. Furthermore, gene expression related to mitophagy were not different between COLD and RT (*PINK1*, *p* = 0.354; *PARK2*, *p* = 0.400; *BNIP3*, *p* = 0.659; *BNIP3* L, *p* = 0.649). See Figure 2.

## 4. Discussion

This study aimed to determine the impact of local cooling on skeletal muscle at rest on gene expression related to mitochondrial homeostasis. To our knowledge, this is the first study to examine mitochondrial biogenesis and mitophagy related genes following local cold application without exercise in humans. Our intention was to separate the commonly found synergistic effect of exercising in ambient cold on *PGC-1α* gene expression [13,14,15,16] while also separating previously observed inhibition/blunting of *PGC-1α* [18] following simultaneous local cold application and exercise. The main findings of this study were that *PGC-1α B* and *GABPA* gene expression increased due to local cold application (1.57-fold and 1.34-fold, respectively). This research represents critical control information to help interpret and contextualize previous and future works investigating the impact of local cold both with and without exercise responses.

As expected with local cooling [19,20], skin temperature (~13 °C) and intramuscular temperature (20.5 °C) at the VL were found to be lower with local cold application. This is distinctly different than more whole body-type cold ambient/environmental exposures [13,15] and cold-water immersion [26] stimuli. Ambient/environmental cold and cold-water immersion stimuli may not adequately concentrate at a singular region to decrease intramuscular temperature to the same magnitude as presented here. Indeed, cold-water immersion for 10 min at 10 °C can decrease intramuscular temperature to ~30 °C [26]. These findings are 9.5 °C higher than observed here despite an identical temperature stimulus. Unlike more whole-body cold ambient/environmental exposures [13,15] and cold-water immersion [26] our longer more localized cooling protocol decreased intramuscular temperature presumably without altering core temperature. Previous research using a similar bilateral leg design observed no changes in core body temperature or mitochondrial gene expression with an ice pack (0 °C) locally placed at the quadriceps after exercise, despite intramuscular temperature decreasing to 26.7 °C [19]. Additionally, the singular laser point temperature and the average temperature of the larger area measured with the thermal camera were similar, demonstrating that these pieces of equipment may be used interchangeably for skin temperature measurements. Further affirming that our intervention yielded homogenous cooling over the target area alone and likely without core temperature changes. Importantly, our intramuscular temperature findings, confirm investigation of the independent effects of a local cold application without heat generating exercise.

It is well-accepted that cooling induces changes in circulatory control for cutaneous vasoconstriction and reduced cutaneous blood flow via *α*-adrenergic reception [27]. We observed no significant cold induced decreases in blood velocity (−3.1%), blood flow (−5.8%), or shear rate (0%), but did observe a significant cold induced decrease in FA diameter (−3.2%). Interestingly, the FA diameter percent decrease is on order with the observed, albeit insignificant, percent decreases in blood velocity and blood flow. Although the observed difference in arterial diameter could be due to structural limb-to-limb differences rather than vasoreactivity response to the temperature stimulus. The magnitude of the observed intramuscular decrease coupled with FA diameter decrease suggests otherwise. For example, it has been shown that reductions in skin temperature of ~5–10 °C induce *α*-adrenergic vasoconstriction and blunted vasodilatory activity [28,29], temperature decreases of which were achieved here. However, previous work has found that local lower extremity cooling does not always impact peripheral hemodynamics [30].

Two promoters, primary and alternate, of *PGC-1α* lead to different isoforms. *PGC-1α A* is derived from the primary promoter, while *PGC-1α B* is derived from the alternate promoter. The primary promoter is largely stimulated by energy state/*AMPK* activation, while *PGC-1a B* is found to be stimulated by adrenergic activity [16,31,32]. Exercise was not used in the present study for *AMPK* stimulation of the primary promoter. As cooling induces cutaneous vasoconstriction and reduced cutaneous blood flow via *α*-adrenergic reception [27]. The local cooling used may have stimulated *PGC-1α B* via the alternate adrenergic stimulated promoter. Cold exposure increases sensitivity to catecholamines regulated by adrenergic coupling to stimulate thermogenesis [33,34]. *PGC-1α B* stimulation due to adrenergic activity in response to the cold may explain the observed *PGC-1α B* increase observed with our local cold intervention.

*GABPA* plays a key role in modulating mitochondrial function and energy homeostasis, helping *PGC-1α* control mitochondrial biogenesis genes [35,36]. *GABPA* is linked to the transcriptional control of mitochondrial related genes through mitochondrial protein import machinery [37]. It functions as a transcription factor that activates expression of key mitochondrial biogenesis genes. *PGC-1α* increases the expression of *GABPA*, *NRF1*, and *ESRRα*, creating a positive feedback loop. Therefore, the increase in *PGC-1a B* may explain the increased expression of *GABPA* as part of this positive feedback. In contrast with the current data, *PGC-1 α* and *NRF1* expression are blunted following local cold application during exercise [18]. Interestingly, no differences in *NRF1* and total *PGC-1 α* were noted in the current study. Similarly, there were no other notable gene expression alterations. These findings suggest that an exercise interaction exists and/or the intramuscular temperature was decreased beyond provoking gene expression alterations. Notwithstanding, the applied biological impact of the observed increases in *PGC-1a B* and *GABPA* following localized cooling is unclear.

## 5. Conclusions

The purpose of this study was to determine the independent effects of localized cooling of the skeletal muscle on mitochondrial related gene expression. While increases in *PGC-1α B* and *GABPA* were found with local cold application, the applied biological impact is difficult to discern from the current study. No other notable gene expression alterations also raise the question if there is an intramuscular temperature decrease threshold of which no changes will occur. This study was also conducted in a healthy young population, and thus the implications in clinical populations may not be translatable. Indeed, it is possible that temperature related stimuli may provide a bigger perturbation in these clinical populations and provide a differential response when compared to healthy young subjects. The current study provides critical control data and helps interpret previous cold interventions incorporating exercise and/or recovery from exercise by removing the heat generating exercise component.

## Figures and Tables

**Table 1:** Probes and Primers used for real-time reverse transcription quantitative PCR.

	Primer 1	Primer 2	Probe
**Reference**			
*B-Actin*	CCTTGCACATGCCGGAG	ACAGAGCCTCGCCTTTG	TCATCCATGGTGAGCTGGCGG
*B2M*	ACCTCCATGATGCTGCTTAC	GGACTGGTCTTTCTATCTCTTGT	CCTGCCGTGTGAACCATGTGACT
*GAPDH*	TGTAGTTGAGGTCAATGAAGGG	ACATCGCTCAGACACCATG	AAGGTCGGAGTCAACGGATTTGGTC
*RPS18*	GTCAATGTCTGCTTTCCTCAAC	GTTCCAGCATATTTTGCGAGT	TCTTCGGCCCACACCCTTAATGG
**Biogenesis**			
*PGC-1α*	AGCCTCTTTGCCCAGATCTT	GGCAATCCGTCTTCATCCAC	AGCTTTCTGGGTGGACTCAAGTGG
*PGC-1α A*	ATGGAGTGACATCGAGTGTGCT	GAGTCCACCCAGAAAGCTGT	AAGACCAGCCTCTTTGCCCAGATC
*PGC-1α B*	CTATGGATTCAATTTTGAAATGTGC	CTGATTGGTCACTGCACCAC	AAGACCAGCCTCTTTGCCCAGATC
*NT-PGC-1α*	TCACACCAAACCCACAGAGA	CTGGAAGATATGGCACAT	AAAGAAGTCCCACACACAGTCGCA
*VEGF*	GCGCTGATAGACATCCATGA	CCATGAACTTTCTGCTGTCTTG	TGCTCTACCTCCACCATGCCAAG
*TFAM*	GCCAAGACAGATGAAAACCAC	TGGGAAGGTCTGGAGCA	CGCTCCCCCTTCAGTTTTGTGTATTT
*ESRRα*	TCTCCGCTTGGTGATCTCA	CTATGGTGTGGCATCCTGTG	TGGTCCTCTTGAAGAAGGCTTTGCA
*NRF1*	GTCATCTCACCTCCCTGTAAC	GATGCTTCAGAATTGCCAACC	ATGGAGAGGTGGAACAAAATTGGGC
*GABPA*	TGGCTTCTGGACTTGGAAC	GACGGTATGCAACAGGACAT	CAATATTAAGACACTGTAACTCAGGAATGGATAATAGCTC
**Mitophagy**			
*PINK1*	GTTGCTTGGGACCTCTCTTG	TGAACACAATGAGCCAGGAG	TGTAAGTGACTGCTCCATACTCCCCA
*PARK2*	GCTTGGTGGTTTTCTTGATGG	TTGAAGCCTCAGGAACAACT	CCTGCTCGGCGGCTCTTTCA
*BNIP3*	CCACTAACGAACCAAGTCAGAC	CATCTCTGCTGCTCTCTCAT	AAAGGTGCTGGTGGAGGTTGTCA
*BNIP3 L*	CAAACATGATCTGCCCATCTTC	TCCTCATCCTCCATCCACAA	TCTCACTGTGACAGCCCTTCGC

Genes include *beta-actin* (*ACTB*), *β2-microglobulin* (*B2M*), *glyceraldehyde-3 phosphate dehydrogenase* (*GAPDH*), *ribosomal protein S18* (*RPS18*), *Peroxisome proliferator-activated receptor gamma coactivator 1 alpha* (*PGC-1α*), *PGC-1α A*, *PGC-1α B*, *NT-PGC-1α*, *vascular endothelial growth factor* (*VEGF*), *transcription factor A mitochondrial* (*TFAM*), *estrogen-related receptor α* (*ESRRα*), *nuclear respiratory factor 1* (*NRF1*), *GA binding protein transcription factor alpha* (*GABPA*), *PTEN-induced putative protein kinase 1* (*PINK1*), *Parkin RBR E3 Ubiquitin Protein Ligase* (*PARK 2*), *Bcl-2/adenovirus E1B interacting protein* (*BNIP3*), and *BNIP3-like* (*BNIP3-L*).

**Table 2:** Arterial hemodynamic results.

	RT	COLD
Blood Velocity (m‧s^–1^)	0.64 ± 0.13	0.62 ± 0.17
Arterial Diameter (cm)	0.62 ± 0.05	0.60 ± 0.05 *
Blood Flow (mL‧min^–1^)	275.0 ± 54.0	259.1 ± 69.0
Shear Rate (s^–1^)	412 ± 27	412 ± 34

* *p* < 0.05 compared to RT. Data are mean ± SD.

## Data Availability

Data presented in this manuscript has not been deposited into public repositories but may be available upon request from the corresponding author.
